# Large language model based simplification of ophthalmological clinical and ancillary results for patient readability

**DOI:** 10.1186/s40942-026-00896-1

**Published:** 2026-07-08

**Authors:** Alexandre Antônio Marques Rosa, Francisco Vinícius Moraes de Souza, José Leandro Nascimento da Silva, Rafael Scherer, Valberto Monteiro Nunes, Leandro Cabral Zacharias, Gustavo Barreto Melo, Taurino dos Santos Rodrigues Neto

**Affiliations:** 1 University of São Paulo (USP). MBA, Pontifical Catholic University of Rio Grande do Sul (PUC-RS), Federal University of Pará (UFPA), São Paulo, Brazil; 2 Federal University of Pará (UFPA), Belém, Brazil; 3 Division of Ophthalmology, Faculdade de Medicina FMUSP, University of São Paulo (USP), Av. Dr Enéas de Carvalho Aguiar, 255, Cerqueira César, 05403-001 São Paulo, Brazil; 4 Retina Prime, Aracaju, Brazil

**Keywords:** Artificial intelligence, Large language models, Prompt engineering, Ophthalmology, Health literacy

## Abstract

**Background:**

Ophthalmological reports are often written at a complexity level that exceeds the reading ability of many patients. Large language models (LLMs) may help simplify these texts, but their performance depends on prompt design and must preserve clinical fidelity. This study evaluated whether different prompting strategies improve the readability and safety of simplified ophthalmological reports.

**Methods:**

We analyzed 443 de-identified reports from a tele-ophthalmology platform, including 280 retinal fundus and 163 ocular ultrasound reports. Each report was simplified by four LLMs (GPT-3.5, GPT-4.0, Gemini, and Copilot) using three prompts: a basic command, a patient-oriented prompt, and a targeted 7th-grade reading level prompt. Readability was measured with the average Readability Grade Level (aRGL). A representative eligible subset of the generated texts was independently evaluated by two third-year ophthalmology residents, and each selected response underwent independent review by both evaluators, with disagreements resolved by a board-certified retina ophthalmologist. Outcomes included factual accuracy, information completeness, and potential for harm.

**Results:**

The original reports were highly complex, with a median aRGL of 13.8 overall, 11.6 for fundus reports, and 15.4 for ocular ultrasound reports. All LLMs improved readability scores to varying degrees. Prompt engineering was a major determinant of performance, and the targeted 7th-grade prompt produced the best results across models. Copilot, Gemini, and GPT-4.0 achieved median aRGL values closest to the recommended patient-facing reading level, while GPT-3.5 showed weaker performance in some comparisons. Clinical validation showed substantial agreement between raters (kappa range, 0.70–0.97). After adjudication, 91% of simplified texts were factually accurate, 86% retained all critical information, and 95% showed no potential for harm.

**Conclusion:**

LLMs can simplify ophthalmological reports while preserving clinical fidelity, but performance depends strongly on prompt specificity. These tools show promise for patient-facing communication, although human oversight remains essential.

## Introduction

Effective patient-provider communication is a cornerstone of clinical practice in ophthalmology, yet it is frequently challenged by the inherent technical complexity of diagnostic and follow-up reports [1, 2]. The specialized terminology and quantitative data integral to ophthalmological assessments, while essential for clinical accuracy, can create significant barriers to patient comprehension. This communication gap undermines the principles of shared decision-making and can negatively impact the patient’s engagement with their own care.

This challenge is magnified for individuals with limited health literacy defined as the capacity to obtain, process, and understand basic health information needed to make appropriate health decisions [3]. Low health literacy is a well-established independent predictor of adverse clinical outcomes across multiple medical fields. Documented consequences include poor treatment adherence, particularly with complex medication regimens for chronic conditions like glaucoma, increased rates of emergency service use, and a subsequent rise in overall healthcare costs [4–6].

The global issue of health literacy is especially pronounced in regions with significant socioeconomic and educational disparities [7]. Brazil, for instance, serves as a compelling case study where high rates of functional illiteracy in the adult population directly impact patients’ ability to interpret essential medical documents [8, 9]. This scenario is not unique; similar challenges are observed worldwide, highlighting a universal need for clearer communication tools in specialized medicine [10].

Text simplification has emerged as a recognized and effective strategy to bridge this communication challenge [11, 12]. Traditionally a manual process, this approach has been revolutionized by the advent of Artificial Intelligence (AI). Specifically, Large Language Models (LLMs) offer a novel and powerful tool for reformulating complex technical texts into more accessible language, demonstrating a capacity to preserve core meaning while adjusting for readability [13, 14–16].

While LLMs are actively investigated for use in general medicine, their specific efficacy and safety in simplifying ophthalmological reports remain to be rigorously evaluated [17]. Key questions remain regarding their ability to accurately convey nuanced clinical information, avoid introducing errors, and produce outputs that are genuinely clearer for patients. The increasing integration of digital health tools further underscores the urgent need to validate these emerging technologies before their widespread adoption [18].

Therefore, this study aimed to quantitatively and qualitatively evaluate the performance of four contemporary LLMs in simplifying technical ophthalmological reports. We assessed the simplified outputs using a combination of established readability metrics and expert clinical validation to determine their potential for safe and effective application in enhancing doctor-patient communication.

## Methods

Study Design and Population: This study employed a quantitative and qualitative, cross-sectional, comparative design to evaluate the text simplification capabilities of four leading Large Language Models (LLMs). The source data consisted of a corpus of 443 de-identified ophthalmological reports, selected from the RedCheck^®^ tele-ophthalmology platform [19], between June 1, 2022, and June 1, 2023. The sample comprised 280 retinal fundus photograph reports and 163 ocular ultrasound reports, representing a typical cross-section of diagnostic imaging documentation in clinical practice. Ethical oversight was conducted in accordance with institutional guidelines, and data privacy was maintained throughout the study.

Large Language Models and Prompt Engineering Strategy: Four commercially available LLMs were selected for evaluation: We evaluated GPT-3.5, GPT-4.0, Gemini 3 Flash Preview (Google, Mountain View, CA, USA), and Microsoft Copilot (Microsoft, Redmond, WA, USA; free web version, December 2025, available at https://copilot.microsoft.com). All interactions were conducted over a short period in a controlled environment, using the same access conditions for all models. This design reduced temporal variability and helped ensure comparability across prompt conditions. To assess the impact of contextual framing on simplification performance, a structured prompt engineering strategy was implemented using three distinct prompts for each report:

- Prompt 1 (Zero-shot, Basic Command): “Simplify this ophthalmological report.”
- Prompt 2 (Persona-based, Audience Context): “I am a patient. Simplify this ophthalmological report.”
- Prompt 3 (Targeted Readability): “Simplify this ophthalmological report to a 7th-grade reading level.”

This multi-prompt approach allowed for the systematic evaluation of model performance under varying degrees of instructional specificity.

Data Processing and Corpus Generation: Each of the 443 source reports was processed by the four LLMs with each of the three prompts, creating a final corpus of 5,316 unique, machine-generated texts (443 reports × 4 LLMs × 3 prompts). A programmatic post-processing pipeline was executed to standardize the outputs for analysis. The cleaning step removed only non-clinical conversational wrappers and was checked against the source output to avoid the loss of clinically meaningful content.

Primary Endpoint: Readability Assessment The primary outcome measure was the change in readability, quantified by the average Readability Grade Level (aRGL). This composite score was calculated for each text using the *Análise de Legibilidade Textual* (ALT) tool [20], which is validated for the Portuguese language. The aRGL integrates the outputs of four established readability indices: the Gunning Fog (GF) index, Flesch-Kincaid Grade Level (FKGL), Automated Readability Index (ARI), and the Coleman-Liau Index (CLI). The use of a composite metric mitigates the known biases of any single index, providing a more robust measure of text complexity. The aRGL score correlates with the U.S. grade level required for comprehension, with a target of 7th–8th grade being the standard recommended by the American Medical Association (AMA) for patient-facing materials [21].

Secondary Endpoint: Clinical validation protocol to assess the clinical fidelity of the simplified texts; a human-in-the-loop validation protocol was implemented. A representative, eligible case selection of the generated texts was independently evaluated by two medical doctors. The evaluators were blinded to the source model, and the order of text review was randomized to minimize assessment bias. Each text was assessed against three objective, dichotomous (pass/fail) criteria:

1. Factual Accuracy: The simplified text introduced no clinically incorrect information or fabrications.
2. Information Completeness: All clinically critical findings from the original report were retained.
3. Potential for Clinical Harm: The simplified text contained no omissions, ambiguities, or alterations that could foreseeably lead to patient misinterpretation resulting in adverse health decisions or behaviors.

Disagreements between the two primary raters were resolved by a senior, board-certified retina ophthalmologist who served as the final adjudicator.

### Statistical analysis

All statistical analyses were performed using Python (v3.9) within a jupyter notebook environment, leveraging the scipy.stats and statsmodels libraries. The non-parametric Wilcoxon signed-rank test was used to compare the aRGL scores of the original reports against their simplified counterparts for each LLM and prompt condition. Inter-rater reliability for the clinical validation was quantified using Cohen’s Kappa coefficient, with the Prevalence and Bias Adjusted Kappa (PABAK) calculated to account for the expected high rate of agreement. The McNemar test was employed to detect systematic directional disagreement between the raters. Post-adjudication proportions of texts meeting the validation criteria were reported with 95% confidence intervals (CI) calculated via the Wilson score method. A two-tailed p-value of < 0.05 was set as the threshold for statistical significance.

## Results

Baseline Readability of Original Reports The 443 original ophthalmological reports exhibited a high level of textual complexity, with a median average Readability Grade Level (aRGL) of 13.8. This corresponds to a late high school or early undergraduate reading level, significantly exceeding the 7th–8th grade level recommended for patient education materials. A notable disparity was observed between modalities: retinal fundus photograph reports had a median aRGL of 11.6, whereas ocular ultrasound reports presented a significantly higher median aRGL of 15.4.

Overall Performance of LLMs in Text Simplification. All LLMs, when prompted appropriately, demonstrated the capability to reduce the readability complexity of the original reports. The median aRGL scores for each model and prompt condition, stratified by report modality, are detailed in Table 1.

GPT-4.0, Gemini (formerly Google Bard), and Copilot (Microsoft Bing) achieved statistically significant reductions in aRGL from baseline across all three prompt conditions and for both report modalities. In contrast, GPT-3.5 failed to produce a significant simplification for ocular ultrasound reports using Prompt 2 and for retinal fundus reports using Prompt 3.

**Fig. 1:**
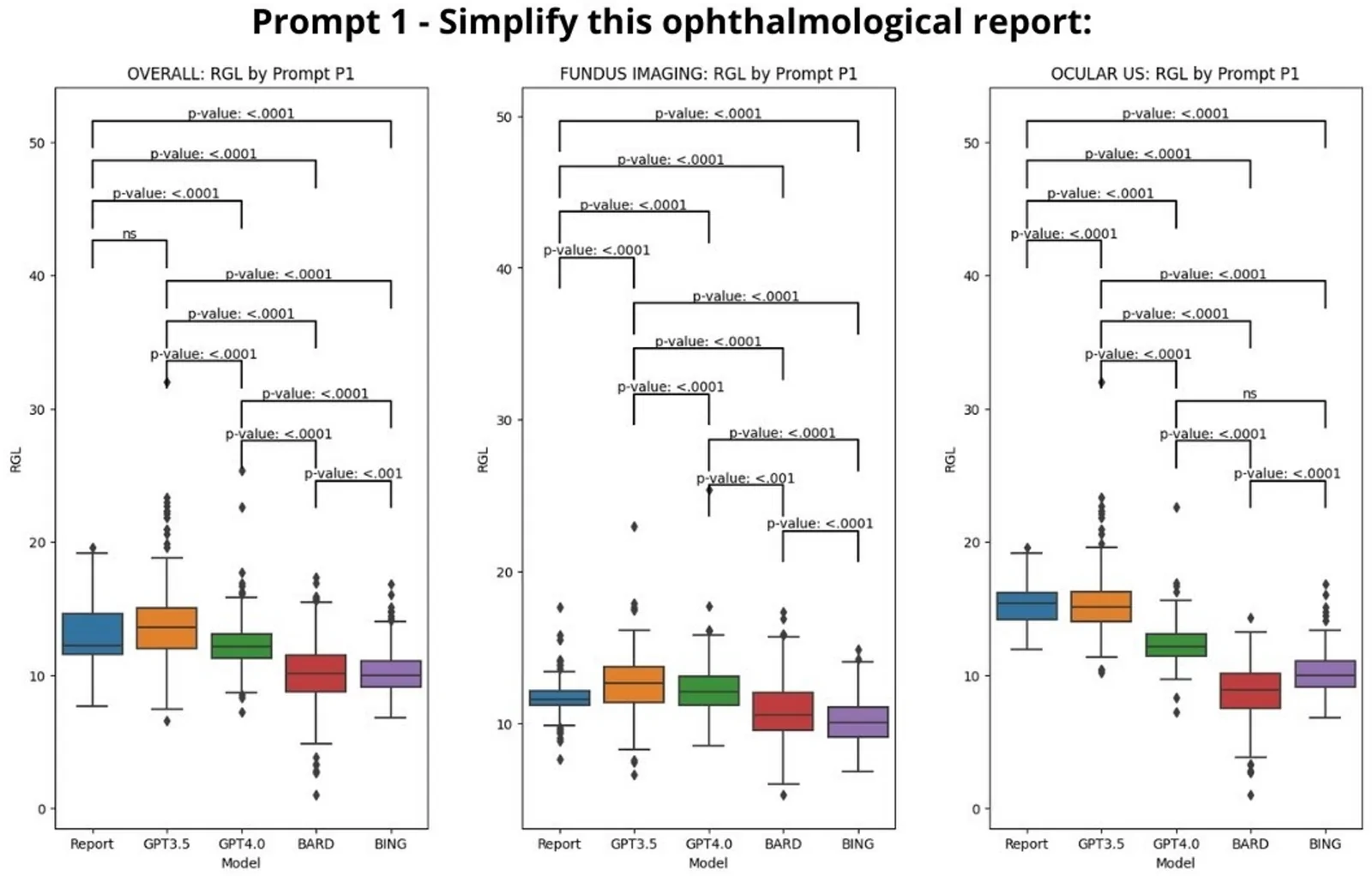
Comparative performance of LLMs using the basic simplification prompt (Prompt 1)

**Fig. 2:**
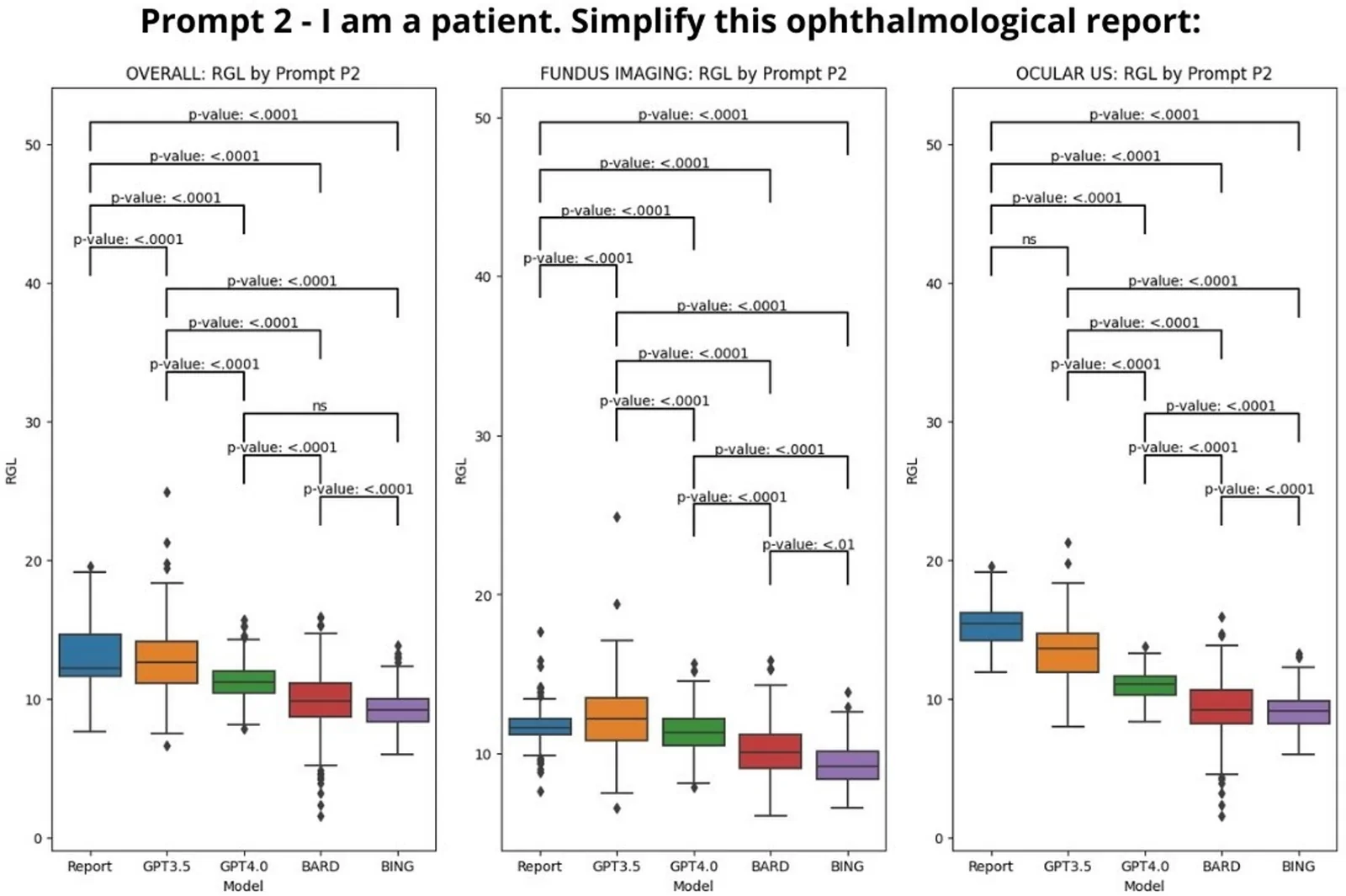
Comparative performance of LLMs using the persona-based prompt (Prompt 2)

**Fig. 3:**
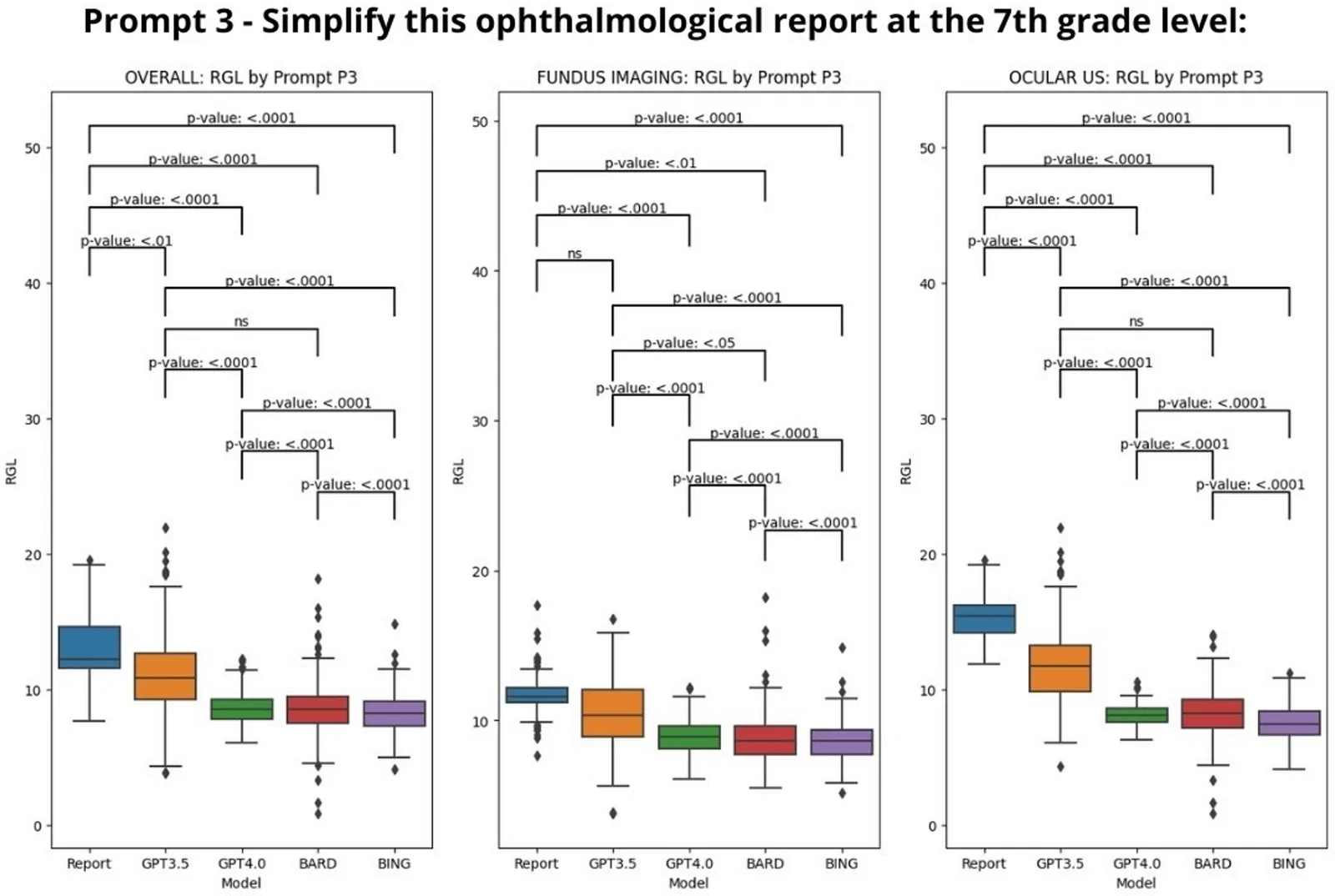
Comparative performance of LLMs using the targeted readability prompt (Prompt 3)

Performance Analysis by Prompt Condition: The instructional prompt was a critical determinant of simplification efficacy:

- Prompt 1 (Basic Command): Gemini and Copilot (median aRGL of 10.1 and 10.0, respectively) significantly outperformed GPT-3.5 and GPT-4.0 (median aRGL of 13.5 and 12.1; p < 0.0001 for both comparisons) (Fig. 1).
- Prompt 2 (Audience Context): Gemini and Copilot maintained their superior performance, yielding significantly lower aRGL scores compared to both GPT models (p < 0.0001 for fundus photography; p < 0.05 for ocular ultrasound) (Fig. 2).
- Prompt 3 (Targeted Readability): This prompt consistently yielded the most effective simplification across all models. Gemini, Copilot, and GPT-4.0 achieved comparable, low aRGL scores (8.5, 8.2, and 8.5, respectively) and significantly outperformed GPT-3.5 (p < 0.05). Copilot achieved the lowest overall median aRGL of 8.2, reaching a median of 7.4 for ocular ultrasound reports, closely aligning with the AMA-recommended level (Fig. 3).

**Fig. 4:**
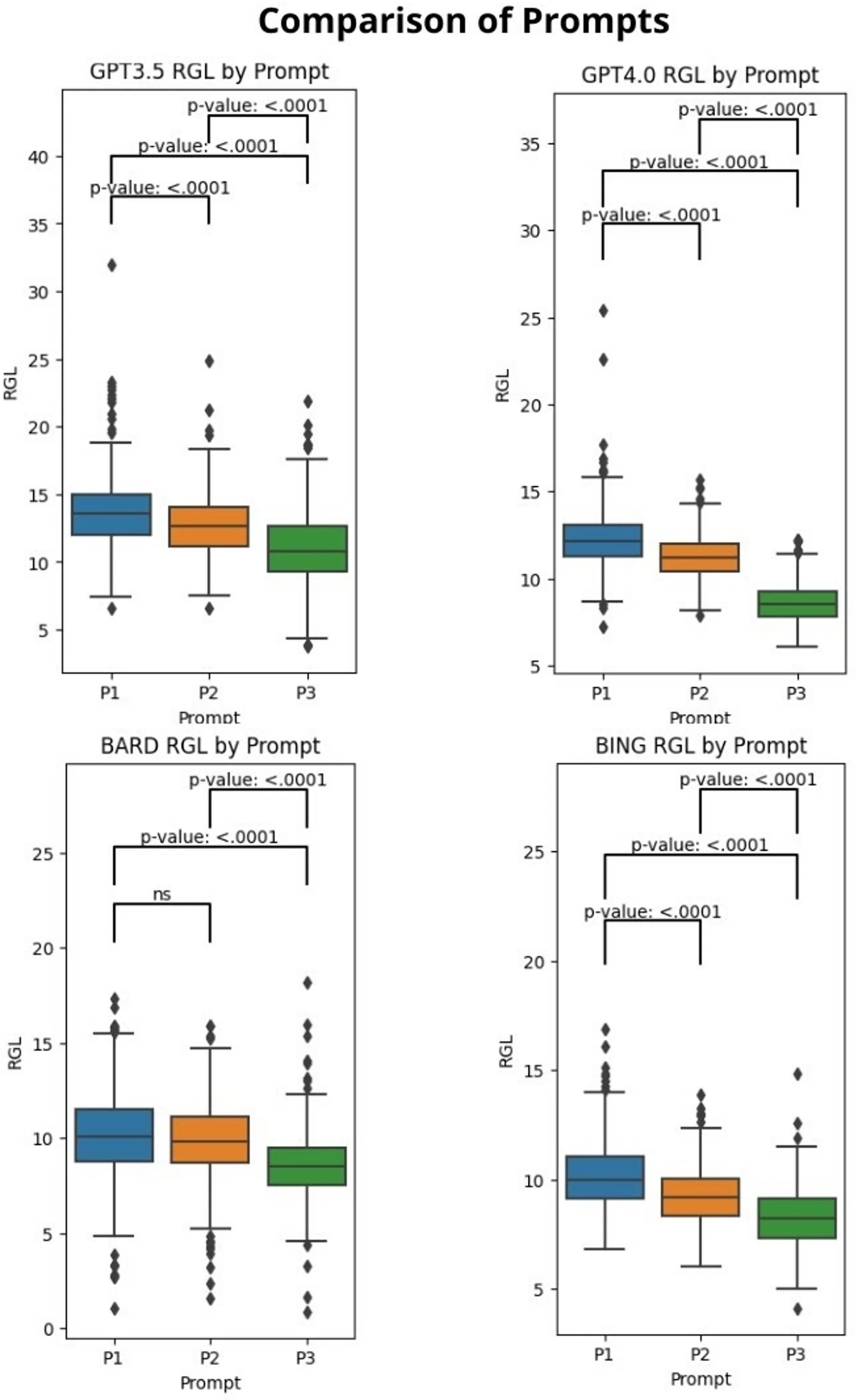
Impact of prompt engineering on text simplification performance across Large Language Models (LLMs)

Inter-Prompt Comparison A direct comparison between prompts revealed that Prompt 3 was unequivocally superior, resulting in the greatest reduction in aRGL for all LLMs (*p* < 0.0001). Furthermore, Prompt 2 (persona-based) was more effective than Prompt 1 (basic) for GPT-3.5, GPT-4.0, and Copilot (*p* < 0.0001), but this difference was not statistically significant for Gemini (*p* > 0.05) (Fig. 4).

Clinical Validation of Simplified Outputs The clinical validation protocol demonstrated high inter-rater reliability, with Cohen’s Kappa coefficients ranging from 0.70 to 0.97, indicating substantial to almost perfect agreement. The McNemar test revealed no systematic directional bias between raters (*p* > 0.05). Following final adjudication by a senior ophthalmologist, the simplified texts were found to have high clinical fidelity:

- Factual Accuracy: 91% (95% CI: 88%–94%) of the simplified texts were deemed factually accurate.
- Information Completeness: 86% (95% CI: 81%–91%) were assessed as having retained all critical clinical information.
- Absence of Potential for Harm: 95% (95% CI: 90%–100%) were judged to be free of content that could pose a potential risk of clinical harm.

**Table 1:** Median average readability grade level (aRGL) of original and simplified ophthalmological reports by Large Language Model (LLM) and prompt condition

Model & Prompt Condition	Overall Median aRGL	Fundus Photo Median aRGL	Ocular US Median aRGL
Original Report (Baseline)	13.8	11.6	15.4
GPT-3.5			
Prompt 1 (Basic)	13.5	12.6	15.1
Prompt 2 (Persona)	12.6	12.1	13.6
Prompt 3 (Targeted)	10.8	9.2	11.7
GPT-4.0			
Prompt 1 (Basic)	12.1	12.1	12.1
Prompt 2 (Persona)	11.2	11.3	11.0
Prompt 3 (Targeted)	8.5*	8.8*	8.1*
Gemini (Google)			
Prompt 1 (Basic)	10.1	10.5	8.8
Prompt 2 (Persona)	9.8	10.0	9.2
Prompt 3 (Targeted)	8.5*	8.6*	8.2*
Copilot (Microsoft)			
Prompt 1 (Basic)	10.0	10.0	9.9
Prompt 2 (Persona)	9.1	9.2	9.1
Prompt 3 (Targeted)	8.2*	8.6*	7.4*

aRGL: Average Readability Grade Level; US: Ultrasound. Prompts: Prompt 1 (Basic: “Simplify this report”), Prompt 2 (Persona: “I am a patient. Simplify…”), Prompt 3 (Targeted: “…to a 7th-grade reading level”). An asterisk indicates that the median aRGL is within or near the AMA-recommended 7th–8th grade level for patient materials.*

The plot compares the average Readability Grade Level (aRGL) of simplified reports generated by four LLMs under three distinct prompt conditions: a basic command (P1), a persona-based prompt (P2), and a prompt with a specific readability target (P3). Statistical comparisons between groups are indicated, where “ns” denotes a non-significant difference (*p* > 0.05).

## Discussion

This study confirms a significant disparity between the textual complexity of standard ophthalmological reports and the average health literacy levels recommended for patient communication. Our baseline analysis revealed that retinal fundus photography and ocular ultrasound reports are written at readability levels corresponding to secondary and tertiary education, respectively, rendering them largely inaccessible to the general patient population. The principal finding of our investigation is that contemporary Large Language Models (LLMs) can effectively reduce this complexity. However, their efficacy is not uniform; it is critically dependent on the specificity of the instructional prompt. We demonstrated that providing explicit context—either by defining the target audience as a patient (Prompt 2) or by setting a specific readability target (Prompt 3)—yields significantly superior simplification compared to a basic command.

Our findings align with and extend the existing literature on health literacy. The decision to target a 7th-grade reading level in Prompt 3 was informed by established guidelines from the American Medical Association (AMA) and the National Institutes of Health (NIH), which advocate for patient materials to be written at a 3rd to 7th-grade level [21, 22]. This recommendation is particularly salient given the documented disparities in reading comprehension globally. Evidence from the Progress in International Reading Literacy Study (PIRLS) indicates that reading proficiency in countries like Brazil lags significantly behind that of the United States [23], underscoring the universal need for simplified medical communication. Our results demonstrate that targeted prompt engineering is a viable strategy to bridge this gap, making LLMs a potentially powerful tool in addressing these long-standing health literacy challenges.

The observed variability in performance among the different LLMs warrants discussion. Discrepancies in simplification efficacy may stem from several factors, including differences in their underlying architectures, training data corpora, and fine-tuning methodologies [24, 25]. For instance, the distinct ability of each model to process specialized medical terminology and numerical data is likely influenced by its exposure to scientific texts during training. It is particularly noteworthy that Copilot (Microsoft Bing), which is built upon an OpenAI framework [26], demonstrated different performance characteristics compared to the native ChatGPT models [27]. Furthermore, the superior performance of GPT-4.0 over its predecessor, GPT-3.5, provides compelling evidence that iterative model updates can lead to tangible improvements in specialized tasks such as medical text simplification.

A primary concern regarding the use of LLMs in clinical settings is the potential for loss of information or the introduction of factual inaccuracies. Our study was designed to rigorously address this issue through a structured clinical validation protocol. The results of this validation are a key strength of our work, revealing that the vast majority of simplified texts maintained factual accuracy (91%) and information completeness (86%), with a very low incidence of content posing a potential for clinical harm (5%). The high inter-rater reliability, confirmed by substantial Kappa coefficients (0.70–0.97) and the absence of systematic bias, substantiates the robustness of our validation methodology. These findings suggest that, with appropriate human oversight, LLMs can function as safe and reliable auxiliary tools for enhancing patient-provider communication.

Nevertheless, this study has several limitations that must be acknowledged. First, the clinical validation, while rigorous, was conducted by two experienced medical doctors and adjudicated by a single senior retina ophthalmologist; a larger panel of specialists could provide more nuanced insights. Second, our cross-sectional design captures a single snapshot in time and does not account for the rapid, continuous evolution of LLM capabilities and the absence of a repeatability analysis to assess whether the same model produces consistent or statistically similar responses across multiple runs. The data was also sourced from a single tele-ophthalmology platform, and future studies should aim to include a more diverse range of report styles and sources. Finally, this study evaluated the simplified text itself, not the direct impact on patient comprehension, a limitation of this study is the absence of direct patient evaluation of the simplified outputs. While medical professionals assessed the readability and clinical accuracy of the generated texts, the ultimate end-users—patients—were not involved in the evaluation process. Future studies should incorporate patient feedback to ensure that the simplified outputs meet the needs and comprehension levels of the target audience.

In conclusion, this study supports the potential utility that LLMs, when guided by specific and context-aware prompts, are powerful tools for simplifying complex ophthalmological reports to a level appropriate for patient comprehension. The clinical validation demonstrates that this simplification can be achieved with a high degree of safety and accuracy, mitigating key risks associated with their use. The immediate clinical implication is that ophthalmologists can leverage these technologies—not as autonomous agents, but as sophisticated “scribes”—to generate patient-friendly summaries of clinical findings, thereby fostering shared decision-making and patient engagement. Future research should focus on longitudinal studies to track model improvements, prospective trials to measure the direct impact on patient outcomes, and the development of best-practice guidelines for integrating these tools safely into clinical workflows.

## Data Availability

No datasets were generated or analysed during the current study.
